# Antiviral effect of 222 nm far-UVC light against human coronavirus and rhinovirus, and murine norovirus using dried inocula

**DOI:** 10.1016/j.infpip.2025.100473

**Published:** 2025-06-14

**Authors:** Ana C. Lorenzo-Leal, Donald Tam, Horacio Bach

**Affiliations:** Division of Infectious Disease, Department of Medicine, University of British Columbia, 2660 Oak Street, Vancouver, BC V6H 3Z6, Canada

Dear Editor,

With the COVID-19 pandemic, different strategies to limit the spread of viruses, such as disinfectants, vaccines, treatments, masking, and ventilation, have been the subject of research approaches. Disinfectants are mainly used to sanitise different spaces or surfaces, inactivating or destroying infectious microorganisms. For example, UV light disinfection systems have been increasingly used to decrease the transmission of pathogens, such as viruses, mainly because of their manageable costs, broader pathogen inactivation, and practical applications [1].

In the present study, the antiviral activity of far-UVC was tested against the positive-stranded RNA human viruses coronavirus 229E (HCoV 229E, ATCC VR-740), human rhinovirus (HRV, ATCC VR-1559), and murine norovirus (MNV, ATCC VR-1937). The cell lines used to replicate the viruses were MRC-5 cells (ATCC CCL-171), H1 Hela (ATCC CLR-1958), and Raw 264.7 (ATCC TIB-71) for the HCoV 229E, HRV, and MNV, respectively. UVX Inc. (Vancouver, Canada) kindly provided a far-UVC fixture with a KrCl far-UVC excimer lamp and a built-in optical filter to remove wavelengths >235 nm and a peak emission of 222 nm.

Plaque assays were performed to assess the titre of the viruses in the study. The experiment consisted of placing 10 μL drop of each virus (HRV, MNV-1, or HCoV 299E) on a sterile 35 x 10 mm glass Petri dish for 10 min to dry at room temperature. The dry virus was placed at a 50 cm distance from a far-UVC light and exposed for 5 min. After far-UVC light exposure, the dry viruses were resuspended in 10 μL of MEM supplemented with 2 % FBS, and then serial dilutions of each virus were done. Positive (no far-UVC treatment) and negative controls (uninfected cells) were used in each plaque assay. Experiments were done in triplicate. Chemical actinometry and radiometry techniques measured the fluence rate and the UV dose delivered to the sample [2] with an average fluence rate of 30.95 ± 0.3 μW/cm^2^ for each experiment, resulting in a dose of 9.31 ± 0.10 mJ/cm^2^ over a 5-minute exposure period.

The far-UVC light tested reduced the three tested viruses by more than 3-log after a 5-minute exposure time (Table 1), and only the HCoV 229E virus was inactivated completely after 5 min of exposure.

**Table 1:** Results from viral exposure to 222 nm far-UVC light

Strain	Exposure time (min)	Sample (PFU/mL)	Control (PFU/mL)	Log_10_ reduction	Dose (mJ/cm^2^)
HCoV 229E	5	<1+E01±<1+E01	3.33E+04±1.75 E+04	3.52^a^	9.31±0.10
HRV	5	3.33E+01±<1+E01	9.30E+04±1.71 E+04	3.45^a^	9.31±0.10
NRV-1	5	8.89E+02±4.71 E+01	2.45E+06±2.45 E+04	3.44^a^	9.31±0.10

PFU, plaque-forming unit.

a *P*-value<0.05 using a t-test analysis.

Previous studies have shown viral inactivation using far-UVC (222 nm) in liquid samples (virus in media and or PBS) [3,4], aerosol [5], or dry inoculum [6]. However, only a few studied the inactivation of HCoV 229E or HRV. To the best of our knowledge, studies have yet to measure the inactivation of MNV. In the case of HRV, we achieved log reductions similar to those in a previous study (24). Still, this achievement was performed using a dose of 19.35 mJ/cm^2^ to achieve a 2.36 log reduction compared to our study, which used a lower dose of 9.31 mJ/cm^2^ to achieve a 3.45 log reduction. Regarding the inactivation of HCoV 229E, a similar dose of 19.42 mJ/cm^2^ was used to achieve a 3.32 log reduction, whereas in our study, 9.31 mJ/cm^2^ achieved a 3.52 log reduction. The differences in the doses used to achieve a similar reduction could be related to the methodology/protocol used to quantify the viruses.

Although the presence of proteins in the viral samples might be considered as not representative of human samples, saliva contains approximately 1% protein [7]. In contrast, FBS in the culturing media contains approximately 90% water and 6%–8% protein, carbohydrates, blood clotting factors, minerals, and hormones. Although 8% is the total percentage of the biomolecules mentioned before, we only use 5–10% in our cell culture, making a maximum of 0.8% protein, which is very similar to the content in saliva [8]. Therefore, we do not consider a significant difference between the human sample (saliva) and the protein content in the media.

Unlike traditional UV technologies (254 nm–280 nm), which have been used widely but are harmful to humans even at lower doses and are therefore limited in their applications, far-UVC light (222 nm) has the added benefit of human skin and eye safety.

## Author contributions

ALL, WT, and HB conceptualized this letter, wrote the letter, and read and approved the final version.

## Ethics statement

None.

## Funding sources

MITACS (Mathematics of Information Technology and Complex Systems) Accelerated Program funded this study, grant No. 020808.

## Conflict of interest statement

None.
